# Effect of Caregivers’ Parenting Styles on the Emotional and Behavioral Problems of Left-Behind Children: The Parallel Mediating Role of Self-Control

**DOI:** 10.3390/ijerph182312714

**Published:** 2021-12-02

**Authors:** Weigang Pan, Baixue Gao, Yihong Long, Yue Teng, Tong Yue

**Affiliations:** 1 Laboratory of Emotion and Mental Health, Chongqing University of Arts and Sciences, Chongqing 402160, China; confessing@163.com (W.P.); gbxpsychology@163.com (B.G.); 2 College of National Culture and Cognitive Science, Guizhou Minzu University, Guiyang 550025, China; yeehsia@163.com; 3 School of Psychology, Northeast Normal University, Changchun 130024, China; tengyue2028@163.com; 4 Faculty of Psychology, Southwest University, Chongqing 400715, China

**Keywords:** left-behind children, caregivers’ parenting styles, dual-mode of self-control, emotional and behavioral problems (EBP)

## Abstract

Childhood is an important period of individual psychological development, and parents’ company and parenting styles are highly significant to children’s personality cultivation and mental health. With the advancement of China’s modernization and urbanization, left-behind children without their parents’ company have become a growing concern. Compared with children raised by their parents, left-behind children are more likely to show social maladaptation and mental health problems. This study explored the mediating effects of left-behind children’s dual mode of self-control between caregivers’ parenting styles and emotional and behavioral problems (EBPs). In this study, 469 left-behind children in senior classes of primary schools were investigated by adopting the caregivers’ parenting styles questionnaire of left-behind children, the dual-mode of self-control scale and the strengths and difficulties questionnaire. This study found that (1) the protective and risk factors for caregivers’ parenting styles not only directly affected EBP, but also affected it through the mediating effect of the dual-mode of self-control, and (2) the mediating effect of the impulsive system was significantly greater than that of the control system. This study confirmed that caregivers’ parenting styles had an important impact on left-behind children’s psychological growth: positive parenting styles not only directly reduced the risk of EBP, but also indirectly improved left-behind children’s mental health by promoting their level of self-control; negative parenting styles directly increased the risk of EBP and indirectly affected left-behind children’s mental problems by enhancing their level of impulsiveness. These findings provide an important basis for reducing the risk of mental health problems and cultivating good personality qualities of left-behind children.

## 1. Introduction

With the advancement of China’s modernization and urbanization, a large number of rural laborers have begun to work in cities or start businesses to change their own economic conditions. However, China’s unique urban–rural separation system has posed economic, educational and medical obstacles, and rural laborers must entrust others to take care of their children while they work in another location, resulting in a large number of left-behind children [1,2]. Left-behind children refer to those who are taken care of by their single parent or other extended family members for more than six months with one parent being away from home [3]; such children are often taken care of by grandparents, uncles, aunts or even older siblings. According to statistics from China’s Ministry of Civil Affairs, by the end of August 2018, there were 6.97 million left-behind children in the country’s rural areas, although that number was down 22.7% from 9.02 million in 2016 [4].

### 1.1. Challenges Faced by Left-Behind Children

Long-term separation from parents and a lack of parental concern and care have exerted a profound impact on the personality development and mental health of left-behind children. Studies have found that left-behind children are more prone to experiencing emotional and behavioral problems (EBPs) than non-left-behind children [5,6]. Specifically, depression, loneliness and other negative self-reaction problems are internalizing problems. Most left-behind children tend to show depression, panic, social anxiety or other types of mental pain [7,8,9]. A meta-analysis also confirmed that left-behind children experience significantly higher academic pressure, loneliness, sadness and despair, and they are more prone to mental health problems than non-left-behind children [10]. Behavioral problems with insufficient control related to the environment, such as hyperactivity, discipline violation and aggression, are externalizing problems. Left-behind children have significantly higher internalization and externalization of EBP and a higher prevalence of specific symptoms than non-left-behind children [11,12]. A meta-analysis of 111 studies (which included a total of 264,967 children, including 106,167 left-behind children) found that parental migration is detrimental to the health of left-behind children and adolescents with no evidence of any benefit [11]. It is worth noting that left-behind children not only suffer from current troubles and pain, but also imperceptibly convey signals of vulnerability and obedience due to EBP [13], consequently making them more vulnerable to types of bullying such as ridicule, abuse and blackmail [14,15]. Such vulnerability further reduces their level of social adaptation [16] and increases their risk of suicide, depression and other mental illnesses [17,18].

### 1.2. Caregivers’ Parenting Styles and the Mental Health of Left-Behind Children

Why does a lack of parental care exert such a large impact on left-behind children? According to ecological systems theory, family is an important microenvironment that affects children’s mental health and the formation of children’s personality and behavior [19]. As one of the core elements of the family environment, parenting styles are a collection of parents’ attitudes, behaviors and emotions that are demonstrated when educating their children and exert an important influence on children’s EBP [20]. Developmental psychologists have pointed out two aspects of parenting styles that are important from childhood to adolescence: parental acceptance/responsiveness and demandingness/control [21]. Acceptance/responsiveness refers to parents’ degree of support and care for their children, and demandingness/control reflects parents’ degree of control or monitoring of their children. Numerous studies have found that warm and responsive parenting styles are consistently associated with positive developmental results, such as secure attachment, good peer relationships, and higher creative abilities [22]. When parents give appropriate requirements and supervision to their children, it is conducive to them forming good behavioral norms of behaviors, possessing a higher level of self-control, and showing fewer selfish, capriciousness and other behavioral problems [23,24]. Due to long-term separation from their parents, left-behind children have difficulty obtaining their parents’ direct care and supervision. Their actual caregivers are usually grandparents, uncles and aunts, and a few left-behind children are even taken care of by elder siblings. These actual caregivers usually provide left-behind children with more material support and life care, but little emotional care and in-depth communication, and they lack adequate guidance in values and behaviors. Therefore, left-behind children are prone to EBP [5,6,7,8].

The compositional structure of left-behind children’s caregivers is complex. Different from parental care, these caregivers show many kinds of parenting styles. Therefore, the influencing mechanism of caregivers’ parenting styles on the personality growth and mental health of left-behind children is complicated. The relationship between caregivers’ parenting styles and the psychological adjustment of left-behind children should be interpreted from multiple perspectives. Most existing studies have explored the influence of a specific parenting style on the psychological development of left-behind children. This approach is not only inconsistent with the actual situation of left-behind children, but also cannot fully explain the underlying mechanisms of their EBP. Some researchers have proposed a two-factor theory to explain the relationship between parenting styles and adolescent problem behaviors [25,26]. The two-factor theory explains the formation mechanism of EBP in terms of both protective and risk factors. This model treats a low level of support and neglectful parenting styles as risk factors for children’s EBP. Studies have found that rejection, control, and severe punishment significantly predict EBP in adolescents [27,28]. Warm and supportive parenting styles are considered protective factors for children’s development [29]. Some studies have confirmed that parental attention and psychological help are significantly positively correlated with children’s mental health and negatively correlated with the probability of children’s EBP [30,31]. Furthermore, the long-term impact of parenting styles on children’s psychosocial development seems to be crucial, even in adulthood [32]. Therefore, this study examines the effects of both protective and risk factors on EBP in left-behind children. We hope to find positive factors that promote the psychological development of left-behind children and avoid or reduce the negative effects of risk factors. Accordingly, this study proposes Hypothesis 1.

**Hypothesis** **1.** *The protective factors of caregivers’ parenting styles can reduce the occurrence of left-behind children’s EBP; the risk factors for parenting styles will hinder the healthy development of left-behind children and lead to or increase the occurrence of EBP.*

### 1.3. Mediating Role of Self-Control

**Figure 1:**
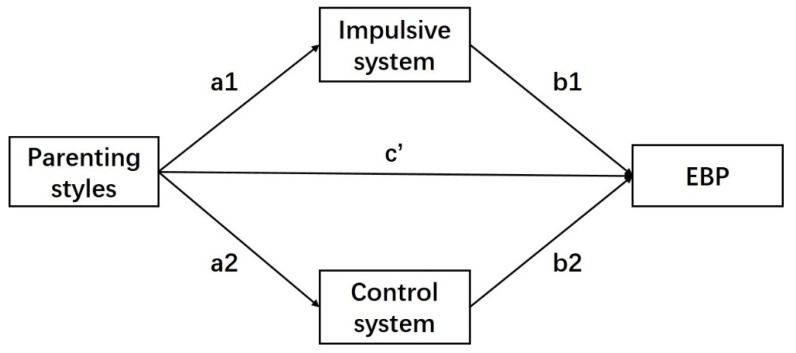
Parallel mediation model for impulsive system and control system. Note: a1, b1, a2, b2 and c’ present the standardized path coefficients.

Parenting styles can affect not only left-behind children’s EBP but also their self-system. According to the self-system process model proposed by Connell and Wellborn, the external environment can influence the individual’s social adaptation level through the individual’s internal self-system [33]. An individual’s internal self-system contains the core subsystem of self-control, and EBP is an important indicator of whether children can adapt well to society. Therefore, parenting styles can affect left-behind children’s self-control and even their EBP by affecting self-control. Numerous studies have confirmed this internal mechanism. First, previous studies have confirmed a strong relationship between parenting styles and self-control. Meldrum’s study found that parental supervision independently predicted higher levels of self-control after controlling for school supervision and peer pressure [34]. A longitudinal study provided evidence of an indirect association between maternal self-control and early childhood self-control through maternal ineffective parenting [35]. However, children with absent fathers had low planning ability, which is an important aspect of self-control [36]. Finkenauer et al. found that reducing manipulative psychological control from parents can help develop children’s self-control [37]. In addition, numerous studies have found that self-control can affect children’s EBP. Self-control is viewed as a personality trait that is associated with a wide range of behaviors [38]. The dual-mode of self-control theory [39] states that self-control is a child’s ability to restrain dominant responses and arouse inferior responses and consists of two different systems: a control system and an impulsive system. A high control system indicates that the individual is high in planning and has a high ability to resist immediate temptation. A high impulsive system, by contrast, indicates that individuals are highly impulsive and poorly able to resist immediate rewards. The success of self-control depends on the antagonistic outcome of the two systems. The dual mode of self-control is a good explanation for mental health problems. High self-control can help individuals resist temptation to eat, maintain healthy dietary habits [40], predict more positive emotions [41], and reduce the risk of substance abuse [42]. Low self-control has been verified as the root of many social and psychological problems, such as substance abuse [43], aggression and antisocial behavior [44]. Further studies have found that self-control is a mediating variable of parenting styles and children’s behaviors and that family intimacy and emotional expression can influence children’s EBP through the mediating effect of self-control [45]. Compared with the traditional definition of self-control, dual mode theory not only further refines the structure of self-control, but also forms a two-dimensional perspective with the dual factors of parenting style, thereby providing a more comprehensive perspective. Since the impulsive system and control system have different neural bases and information processing, this study examined the parallel mediating effect of self-control. We propose Hypothesis 2. See Figure 1.

**Hypothesis** **2.** *The dual mode of self-control has a mediating effect on parenting styles and EBP.*

Although there have been many studies on the psychological development of left-behind children, few studies have explored the influence of the parenting styles of actual caregivers on personality traits (such as self-control) and EBP. Adolescence is a sensitive period for children’s psychological development during which left-behind children will encounter many problems that need to be addressed. This study took left-behind children (grades 5–6) in primary schools, who are about to enter puberty or have just entered adolescence, as participants and studied EBP within the framework of the dual mode of self-control theory.

## 2. Methods

### 2.1. Participants

The study recruited participants in Chongqing, one of the cities with a large number of left-behind children in Southwest China. Questionnaires were administered in whole classes in elementary schools in different areas of Chongqing. The survey was conducted by psychological researchers under the organization of the teacher in charge of each class. A total of 900 children participated in the questionnaire survey. There were 22 classes in total, including 12 classes in grade 5 and 10 classes in grade 6. Among them, 469 left-behind children were identified through self-reporting. Data from these left-behind children were used as the data analysis for this study. Fifty-five participants were excluded because they did not complete the survey or understand the instructions, resulting in a final sample of 414, including 202 boys and 212 girls. The mean age was 11.95 years old (SD = 0.88, full range of 10–14 years). Among the left-behind types, grandparents’ parenting (maternal grandfathers, maternal grandmothers, grandfathers, grandmothers, etc.) accounted for the largest proportion, at 52.80%. Single parenting (one parent’s parenting) accounted for 41.75%. Elder generations’ parenting (uncles, aunts, etc.) comprised 1.72%, and other types accounted for 2.21%. Approximately 42.71% of children had been left behind for 1–2 years; 15.64% of children had been left behind for 3–4 years; 13.67% of children had been left behind for 5–6 years; and 23.03% of children had been left behind for more than 6 years.

### 2.2. Measures

#### 2.2.1. Caregivers’ Parenting Styles Questionnaire of Left-Behind Children

This study adopted the Caregivers’ Parenting Styles Questionnaire of Left-behind Children [46] to investigate parenting styles. Different from general parenting styles questionnaires, this questionnaire was specially designed for left-behind children, fully considered various complicated parenting styles conditions, and uniformly used the concept of “caregivers” to represent both parents and other important caregivers of left-behind children. The questionnaire contained six dimensions: rejection, partiality, severe punishment, emotional care, material care and excessive intervention. Rejection refers to the caregiver’s negative and rejecting attitude toward the child. Partiality reflects differences in caregivers’ preferences for children in the home. Severe punishment reflects the severity of discipline and the severity of punishment for negligence. Emotional care and material care represent the caregivers’ emotional and financial concerns for left-behind children, respectively. Finally, excessive intervention refers to strict requirements and restrictions on the social activities of left-behind children. This questionnaire has a total of 31 items, scored according to “1—never”, “2—occasionally”, “3—often” and “4—always”, where a higher score indicates a higher degree of involvement in a certain parenting style. Sample items include “They always complain to others that I am very difficult to discipline”, “I can feel their support when I am nervous or upset”, and “They do not care what I think”. This questionnaire, originally developed in a Master’s Thesis [41], has been used in many studies and has proven to be effective in measuring the parenting styles of caregivers of left-behind children [47,48]. The total internal consistency coefficient of this scale in this study was 0.90.

#### 2.2.2. Dual-Mode of Self-Control Scale

Self-control was measured by the Dual-Mode of Self-Control Scale (DMSC) [49]. This questionnaire was developed based on the dual system theory of self-control [39]. Self-control consists of two different systems: the impulsive system and control system. The impulse system reflects the individual’s impulsive characteristics, whether they are easily distracted, and if they have difficulty delaying gratification. The control system reflects the individual’s ability to consider long-term interests when making decisions. The revised Chinese version of the DMSC [50] has been shown to be reliable and valid. The revised scale has 21 items covering 5 dimensions: impulsivity, distractibility, poor delay of gratification, problem solving and future time perspective. The first three dimensions belong to the impulsive system, while the last two dimensions belonged to the control system. The scale adopted a 5-point Likert scoring method, scored from “1 (very inconsistent)—5 (very consistent)”. Sample items include “I often do what I think, without respect to the results”, “I have a habit of leaving decisions until tomorrow”, and “I often make plans in advance because I can decide my future”. The impulsive system is calculated according to the total score of impulsivity, distractibility, and poor delay of gratification; higher scores indicate poorer self-control. The control system is calculated according to the total score of problem solving and future time perspective; higher scores indicate better self-control. The Cronbach’s α coefficient of the total scale was 0.88 in the present study.

#### 2.2.3. Strengths and Difficulties Questionnaire

EBP was measured by the Strengths and Difficulties Questionnaire (SDQ) [51], which is mainly used to evaluate the mental health status of children and adolescents. The revised Chinese student version has good reliability and validity [52] and is applicable to children and adolescents aged 11–16. This questionnaire has 25 items, each of which is scored on a 3-level scale of “0—not true”, “1—somewhat true”, and “2—completely true”. The SDQ includes five factors: emotional symptoms, conduct problems, hyperactivity, peer problems and prosocial behavior. The total difficulty score is the sum of the first four factors; higher total scores indicate more serious EBP. Sample items include “I am often worried and preoccupied” and “I am often unhappy, heavy-hearted or in tears”. The prosocial behavior factor is the strength factor and was independent of other difficulty factors; higher scores indicate a higher degree of prosocial behavior. Sample items are “I try to be kind to others and care about their feelings” and “I will be kind to children younger than me”. The Cronbach’s α coefficient of the total questionnaire was 0.82. In this study, the difficulty score was used to evaluate the EBP of left-behind children.

### 2.3. Statistical Analysis

The data were input into EpiData and analyzed with the SPSS 23.0 software. Correlation analysis and regression analysis were used to test the relationships among parenting styles, self-control, and EBP. The bootstrap method was used to test the mediating effect [53]. Psychometrics indicate that parenting styles, self-control and EBP should be analyzed as latent variables. Therefore, we attempted to conduct a structural equation model (SEM) in AMOS 23, with maximum likelihood estimation used to estimate parameters. The results showed that the model fix indices were acceptable. Previous studies have shown that the latent variable models provided coefficient estimates that were typically more accurate (and appreciably larger, for paths a, b, and a * b) than those produced by an observed variable approach that ignored measurement error [54,55]. However, the precision of the latent variable models was noticeably reduced relative to observed variable models [54]. Therefore, we tried another approximate approach and treated these variables as observed variables. SPSS Macro PROCESS (PROCESS is written by Andrew F. Hayes, http://www.Afhayes.com (accessed on 12 July 2021)) was used to test the mediating effect of self-control on caregivers’ parenting styles and EBP of left-behind children. The results showed that the two methods yielded similar results (see the Supplementary Material for PROCESS results).

## 3. Results

### 3.1. Common Method Bias

To avoid common method bias, the questionnaire survey was carried out in strict accordance with the psychometric requirements. The survey was anonymous and was administered at different times and locations. Some questionnaire items were scored in reverse. Harman’s one-factor test was used to check for common method bias [56]. The results showed that a total of 20 factors had eigenvalues greater than one, and the first principal factor explained 18.51% of the variance (<40%), indicating that the present study had no serious common method bias [57].

### 3.2. Correlations of Caregivers’ Parenting Styles, the Dual-Mode of Self-Control and EBP

Pearson correlation analysis was used to examine the relationships among caregivers’ parenting styles and the self-control and EBP of left-behind children. As mentioned above, the purpose of this study was to explore the impact of caregivers’ parenting styles on the behavioral problems of left-behind children from multiple perspectives and to understand the different roles of protective and risk factors of parenting styles on children’s behavior. Therefore, we analyzed the relationship among the multiple factors of parenting styles and other variables. Furthermore, this study was based on the dual model perspective of self-control, so the impulsive system and control system of self-control were analyzed separately. Overall EBP were used as indicators of the psychological adaptation of left-behind children. The results showed that rejection, partiality, severe punishment, emotional care and material care were significantly correlated with self-control, as well as EBP (ps < 0.01). Specific results are shown in Table 1.

### 3.3. The Mediating Effect of Caregivers’ Parenting Styles on Self-Control and EBP

The bias-corrected percentile bootstrap method was used to test the mediating effect [58]. The maximum likelihood estimation was used to estimate the model fit indices. The results showed that the fit indices were acceptable (see Table 2 for details).

**Figure 2:**
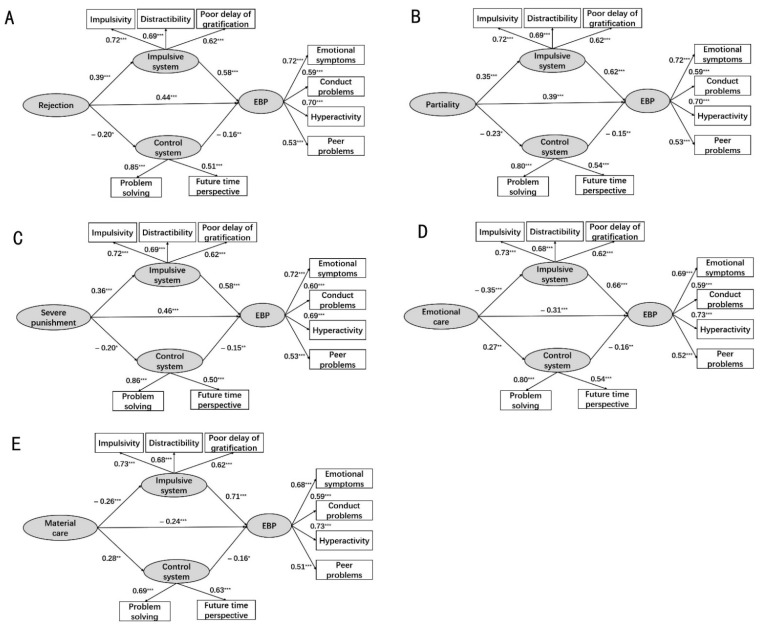
The mediating effects model for impulsive system and control system. (**A**–**E**) show the effects of rejection, partiality, severe punishment, emotional care and material care on EBP, respectively. Standardized path coefficients (β) are presented. Note: *: *p* < 0.05; **: *p* < 0.01; ***: *p* < 0.001.

The mediating effects analysis showed that the bootstrap 95% confidence interval did not include 0 for five caregivers’ parenting styles (rejection, partiality, severe punishment, emotional care and material care), indicating that the impulsive system and control system had significant mediating effects on these parenting styles and EBP (see Figure 2).

Bayes estimation resampling method suggested by Chen and Hung [59] was used in order to compare the mediating effect sizes between impulsive system and control system. Results showed that the mediating effect of the impulsive system of self-control was significantly larger than that of the control system. See Table 3 for details.

## 4. Discussion

This study investigated the influence of caregivers’ parenting styles on the EBP of left-behind children in senior grades of primary schools and analyzed the mediating effect of self-control. The results showed that rejection, partiality, severe punishment, emotional care and material care in caregivers’ parenting styles were correlated with self-control and EBP. The mediating effect analysis further revealed that the protective and risk factors for caregivers’ parenting styles could not only directly affect the EBP of left-behind children, but also indirectly affect EBP through the mediating effect of the dual mode of self-control.

### 4.1. The Relationships among Caregivers’ Parenting Styles, Self-Control and EBP

This study found that material care and emotional care were positively correlated with the control system, but negatively correlated with the impulsive system and EBP. Rejection, partiality and severe punishment were negatively correlated with the control system, but positively correlated with the impulsive system as well as EBP, which was consistent with previous results [37,60]. The first thing that parents of left-behind children should solve in a migrant work environment is the family economic situation, which has been found to be an important factor affecting the social adaptation of left-behind children [61]. Material support would help left-behind children feel emotional warmth, which is a tender way of communicating between parents and children (e.g., emotional care). It has been shown that warm and authoritative parenting styles are correlated with positive adaptation in adolescents [62,63,64], and good parent–child relationships and communication prevent children from experiencing depression and other negative effects [65]. These positive parenting styles create a good environment for children to learn to resist temptation, thus improving their self-control ability and reducing the occurrence of EBP. However, negative parenting styles such as rejection, partiality and severe punishment, as well as the impulsive system of self-control, significantly predicted the occurrence of EBP [66,67]. As their parents leave home to work, the family structures of left-behind children are incomplete. Thus, rejection, partiality and other negative parenting styles reduce the family function of left-behind children, and it is difficult for them to feel close attachment relationships and have sufficient social support. Such situations produce negative emotions such as sadness, anger and hatred or bad behaviors such as learning weariness and discipline violation, which are not conducive to children’s healthy growth. Therefore, positive and warm parenting styles are essential for left-behind children to develop a healthy self-control system and to prevent and intervene in EBP. These results supported Hypothesis 1.

Notably, this study found no significant correlation among excessive intervention, the dual mode of self-control and EBP. One reason may be that it is unclear whether excessive intervention is a positive or negative parenting style for left-behind children. Studies of interviews with left-behind children have found that most left-behind children are eager to be cared for by their parents; sufficient care from other caregivers will impact them, but many caregivers regard protection and excessive intervention as ways to avoid taking responsibility, seeking no merit, but also no fault [46,68]. Excessive intervention from care tends to be a positive parenting style, while intervention from buck-passing tends to be a negative parenting style. For left-behind children, different caregivers may have different motivations, or the same caregiver may simultaneously have two types of motivation. Therefore, it is difficult to clarify the influence of excessive intervention on the psychological development of left-behind children.

### 4.2. Mediating Effect of the Dual Mode of Self-Control

This study found that caregivers’ parenting styles could not only directly affect the EBP of left-behind children, but also indirectly affect EBP through the dual mode of self-control. Moreover, both positive and negative parenting styles tended to affect EBP more through the mediating effect of the impulsive system. Previous studies have also found that children with low self-control were more likely to be positively influenced by their parents than those with high self-control [60]. This phenomenon may be correlated with the different neural bases of the dual mode of self-control [69]. The impulsive system activates the prefrontal cortex-amygdala circuit, which is derived from the automatic emotional and behavioral connections of long-term experience and learning, while the control system activates the middle frontal lobe-striatum circuit, which includes deliberate evaluation and inhibition criteria [70]. In other words, the impulsive system is involved mainly in emotional motivation, while the control system is more correlated with executive function. Both internalizing problems of negative emotion and externalizing problems such as impulse, discipline violation and aggression with insufficient control are more closely related to the emotional system. A study by Sternberg [71] found that children with externalizing problems were characterized by a high degree of anger, substantial impulsivity and low self-control, while children with internalizing problems were characterized by a high degree of sadness, substantial impulsivity and low self-control. Moreover, the results of a high degree of anger in children with internalizing problems were considered unreliable [72] because they were often over-controlled and did not express anger, so the degree of anger was often underestimated. Other studies have asserted that the key to solving internalizing problems is cognitive training [73] to prevent internalizing problems by practicing distraction from negative feelings, thoughts or environmental cues [71,74,75]. However, in this study, the difficulty score of the SDQ was the sum of emotional symptoms, conduct problems, hyperactivity, and peer problems. Among those, only emotional symptoms belonged to internalizing problems. In the present study, when emotional symptoms were analyzed separately instead of EBP in the mediating effect analysis, the results showed that the mediating effect of the impulsive system was still greater than that of the control system, indicating that EBP was indeed more closely correlated with the impulsive system. In addition, neuroimaging studies have shown that the operation of the control system consumes cognitive resources [76], whereas the activation of the impulsive system is related to the reward system and is more automatic [77]. Compared with the impulsive system, which preserves cognitive resources, the activation of the control system relies highly on cognitive resources and individual resource mobilization, which may be the reason why the mediating effect of the impulsive system was greater than that of the control system. These results verified Hypothesis 2.

These results suggested that positive parenting styles (such as material and emotional care) offered by caregivers to left-behind children and negative parenting styles (such as rejection and severe punishment) avoided or reduced by caregivers could maintain children’s mental health more through reducing the impulsivity level rather than improving self-control ability (such as problem-solving ability). This finding may be congruent with the fact that left-behind children in senior grades of primary schools are in or about to enter adolescence. Studies have argued that adolescent children have a high sense of self, face greater pressure and have more mental conflicts [78]. This finding suggests that educational interventions for left-behind children in senior grades of primary schools should give more consideration to their psychological development characteristics and prioritize positive psychological counseling.

### 4.3. Limitations

The present study had several limitations that should be recognized. First, this study is essentially a cross-sectional study, and no causal inferences can be drawn. Therefore, future prospective studies could be adopted to assess the effects of parenting styles on EBP through a longitudinal follow-up. Experimental intervention studies can also be conducted in areas where conditions permit. Second, we discussed only the parallel mediating effect of the dual mode of self-control. Hofmann et al. believed that the results of self-control depended not only on the antagonistic relationship of the impulsive system and control system, but also on the interaction of state and trait variables related to self-control, such as peer relationships, emotions and values [39]. From the perspective of ecological system theory [19], children are not merely passively affected by parenting styles; rather, their personality traits (such as optimism and resilience) also affect caregivers’ parenting styles and have a buffering effect on the adverse effects of negative parenting styles. Peer relationships are also very important in children’s development. Can peer relationships compensate, to some extent, for the lack of parent–child relationships? Future studies need to examine these aspects in depth. Furthermore, the control system of left-behind children had a weak mediating effect between caregivers’ parenting styles and EBP. The activation of the control system required more resources. Is this result correlated with the psychological capital and physical health of left-behind children? Qualitative research methods, such as in-depth interviews and diary analysis might provide a new understanding of the problem.

## 5. Conclusions

Despite the limitations stated above, this study verified that caregivers’ parenting styles exerted an important impact on left-behind children’s psychological growth. Specifically, rejection, partiality, severe punishment, emotional care and material care in caregivers’ parenting styles were correlated with self-control and EBP. Furthermore, the protective and risk factors for caregivers’ parenting styles could not only directly affect the EBP of left-behind children, but also indirectly affect EBP through the mediating effect of the dual mode of self-control.

These findings helped us advance our understanding of the psychological development of left-behind children and hold some implications for practice. Unlike non-left-behind children, left-behind children face many practical problems that need to be addressed. Parents who work outside the home should first provide their children with enough material security to meet their daily needs. It is more important to give left-behind children emotional warmth and moderate discipline. Teachers can organize sports and games to provide targeted training for left-behind children’s concentration. Any way to make left-behind children feel loved and cared for at all times, even though their parents are not around, is beneficial to their psychological development.

## Figures and Tables

**Table 1:** Descriptive statistics and correlations.

	1	2	3	4	5	6	7	8	9
1 Rejection	-								
2 Partiality	0.74 **	-							
3 Severe punishment	0.76 **	0.75 **	-						
4 Emotional care	−0.68 **	−0.64 **	−0.64 **	-					
5 Material care	−0.50 **	−0.49 **	−0.48 **	0.68 **	-				
6 Excessive intervention	0.16 **	0.13 **	0.17 **	0.01	−0.04	-			
7 Impulsive system	0.29 **	0.25 **	0.27 **	−0.27 **	−0.15 **	0.06	-		
8 Control system	−0.15 **	−0.16 **	−0.13 **	0.19 **	0.15 **	0.01	−0.39 **	-	
9 EBP	0.54 **	0.48 **	0.53 **	−0.47 **	−0.32 **	0.11 **	0.58 **	−0.34 **	-
M	1.69	1.65	1.64	2.86	2.62	2.36	2.18	3.28	0.67
SD	0.63	0.61	0.58	0.83	0.66	0.57	0.74	0.77	0.33

Note: **: *p* < 0.01. M = Mean, SD = Standard Deviation.

**Table 2:** Model fit indices.

	x^2^	(*p*)	df	x^2^/df	NFI	NNFI	CFI	GFI	AGFI	RMSEA
Rejection	239.506	0.000	72	3.327	0.860	0.870	0.897	0.920	0.883	0.075
Partiality	253.739	0.000	72	2.985	0.863	0.880	0.903	0.922	0.890	0.069
Severe punishment	232.888	0.000	72	3.235	0.866	0.876	0.902	0.924	0.889	0.074
Emotional care	251.192	0.000	72	3.489	0.875	0.882	0.906	0.920	0.883	0.078
Material care	229.116	0.000	72	3.183	0.847	0.859	0.889	0.925	0.891	0.073

**Table 3:** Results of multiple mediating effects.

Types of Parenting Styles	Variables	Point Estimate	Bootstrap SE	Bootstrapping 95% Bias-Corrected Interval	
				Lower	Upper
Rejection	a1b1	0.142	0.033	0.088	0.221
	a2b2	0.020	0.013	0.003	0.055
	a1b1 − a2b2	0.121	0.035	0.066	0.206
	a1b1 + a2b2	0.162	0.036	0.101	0.244
Partiality	a1b1	0.120	0.030	0.068	0.184
	a2b2	0.019	0.012	0.002	0.053
	a1b1 − a2b2	0.101	0.032	0.048	0.176
	a1b1 + a2b2	0.139	0.139	0.079	0.206
Severe punishment	a1b1	0.124	0.026	0.079	0.182
	a2b2	0.018	0.011	0.003	0.049
	a1b1 − a2b2	0.107	0.029	0.056	0.171
	a1b1 + a2b2	0.142	0.028	0.091	0.204
Emotional care	a1b1	−0.270	0.042	−0.105	−0.172
	a2b2	−0.085	0.019	−0.006	−0.032
	a1b1 − a2b2	−0.247	0.046	−0.066	−0.140
	a1b1 + a2b2	−0.203	0.046	−0.308	−0.128
Material care	a1b1	−0.119	0.041	−0.208	−0.050
	a2b2	−0.028	0.020	−0.081	−0.004
	a1b1 − a2b2	−0.091	0.047	−0.196	−0.016
	a1b1 + a2b2	−0.147	0.044	−0.247	−0.073

Note: SE: Standard Error; a1b1: the indirect effect of caregivers’ parenting styles on EBP by impulsive system; a2b2: the indirect effect of caregivers’ parenting styles on EBP by control system.

## Data Availability

The datasets used and analyzed in the study are available from the corresponding author on reasonable request.

## Supplementary Materials

The following are available online at https://www.mdpi.com/article/10.3390/ijerph182312714/s1, Figure S1: The mediating effects model for impulsive system and control system. (A–E) show the effects of rejection, partiality, severe punishment, emotional care and material care on EBP, respectively. Standardized path coefficients (β) are presented. Table S1: Regression analysis of parenting styles on self-control and EBP in left-behind children. Table S2: The mediating effect of self-control on parenting styles and EBP in left-behind children.
