# Supplementary material for: Effect of Caregivers’ Parenting Styles on the Emotional and Behavioral Problems of Left-Behind Children: The Parallel Mediating Role of Self-Control
Source: Int J Environ Res Public Health. 2021 Dec 2;18(23):12714. doi: 10.3390/ijerph182312714 (PMC8657231; doi:10.3390/ijerph182312714)
Supplement: Supplementary file 1 [file ijerph-18-12714-s001.zip › ijerph-1437890-supplementary.pdf]

The mediating effect of caregivers' parenting styles on self-control and EBP

The bias-corrected percentile bootstrap method was used to test the mediating effect. After controlling variables, such as gender, age, left-behind time and left-behind types, the results showed that rejection, partiality and severe punishment in caregivers' parenting styles significantly positively predicted EBP (β = 0.40, p < 0.001; β = 0.35, p < 0.001; β = 0.40, p < 0.001) and the impulsive system of self-control (β = 0.29, p < 0.001; β = 0.24, p < 0.001; β = 0.26, p < 0.001) and significantly negatively predicted the control system (β = -0.14, p < 0.01; β = -0.14, p < 0.01; β = -0.10, p < 0.05). Emotional care and material care significantly negatively predicted EBP (β = -0.33, p < 0.001; β = -0.23, p < 0.01) and the impulsive system of self-control (β = -0.27, p < 0.001; β = -0.14, p < 0.01) and significantly positively predicted the control system of self-control (β = 0.18, p < 0.001; β = 0.13, p < 0.01). However, the regression effect of excessive intervention was not significant. See **Table S1** for details.

Table S1. Regression analysis of parenting styles on self-control and EBP in left-behind children.

Regression Equation		Fitting Indices		Significance	
Outcome Variables	Predictor Variables	R^2	*F*	*Beta*	*t*
Impulsive system	Rejection	0.10	8.66***	0.29	6.15***
Control system	Rejection	0.06	5.30***	-0.14	-2.81**
EBP	Rejection	0.50	57.97***	0.40	10.81***
	Impulsive system			0.42	10.63***
	Control system			-0.10	-2.65**
Impulsive system	Partiality	0.07	6.13***	0.24	5.03***
Control system	Partiality	0.06	5.42**	-0.14	-2.91**
EBP	Partiality	0.47	51.10***	0.35	9.49***
	Impulsive system			0.45	11.33***
	Control system			-0.09	-2.36*
Impulsive system	Severe punishment	0.08	6.95***	0.26	5.42***
Control system	Severe punishment	0.05	4.49***	-0.10	-2.01*
EBP	Severe punishment	0.50	57.55***	0.40	10.70***
	Impulsive system			0.43	10.91***
	Control system			-0.12	-2.97**
Impulsive system	Emotional care	0.08	7.47***	-0.27	-5.65***
Control system	Emotional care	0.07	6.49***	0.18	3.68***
EBP	Emotional care	0.45	48.16***	-0.33	-8.53***
	Impulsive system			0.45	11.11***
	Control system			-0.09	-2.10*
Impulsive system	Material care	0.03	2.72*	-0.14	-2.91**
Control system	Material care	0.06	5.04***	0.13	2.58**
EBP	Material care	0.41	39.62***	-0.23	-5.85**
	Impulsive system			0.51	12.11***
	Control system			-0.10	-2.26*

The mediating effects analysis showed that the bootstrap 95% confidence interval did not include 0 for the first five caregivers' parenting styles (rejection, partiality, severe punishment, emotional care and material care), indicating that the impulsive sys-tem and control system had significant mediating effects on these parenting styles and EBP. Furthermore, the

mediating effect of the impulsive system of self-control was significantly larger than that of the control system. See **Table S2** and **Figure S1** for details.

Table S2. The mediating effect of self-control on parenting styles and EBP in left-behind children.

Types of parenting styles		Indirect effect	Boot SE	Boot CI LL	Boot CI UL	Relative mediating effect
Rejection	Total indirect effect	0.07	0.01	0.04	0.10	25.46%
	Impulsive system	0.06	0.01	0.04	0.09	22.86%
	Control system	0.01	0.00	0.01	0.02	2.60%
	I-C	0.06 [a]	0.01	0.03	0.09	/
Partiality	Total indirect effect	0.07	0.02	0.04	0.10	25.72%
	Impulsive system	0.06	0.02	0.03	0.09	22.96%
	Control system	0.01	0.00	0.01	0.02	2.76%
	I-C	0.05	0.02	0.02	0.09	/
Severe punishment	Total indirect effect	0.07	0.01	0.04	0.10	23.81%
	Impulsive system	0.06	0.01	0.04	0.09	21.61%
	Control system	0.01	0.00	0.01	0.02	2.17%
	I-C	0.06	0.01	0.03	0.09	/
Emotional care	Total indirect effect	-0.05	0.01	-0.08	-0.03	29.55%
	Impulsive system	-0.05	0.01	-0.07	-0.03	26.32%
	Control system	-0.01	0.00	-0.01	-0.01	3.28%
	I-C	-0.04	0.01	-0.07	-0.02	/
Material care	Total indirect effect	-0.05	0.01	-0.08	-0.03	34.86%
	Impulsive system	-0.05	0.01	-0.07	-0.03	31.06%
	Control system	-0.01	0.00	-0.01	-0.01	3.87%
	I-C	-0.04	0.01	-0.07	-0.02	/

Note：SE: Standard Error; CI: Confidence Interval; LL: Lower Limit; UL: Upper Limit; I-C: Impulsive system minus Control system; a: Since only three decimal places are retained, the value of I - C is close to 0.06.

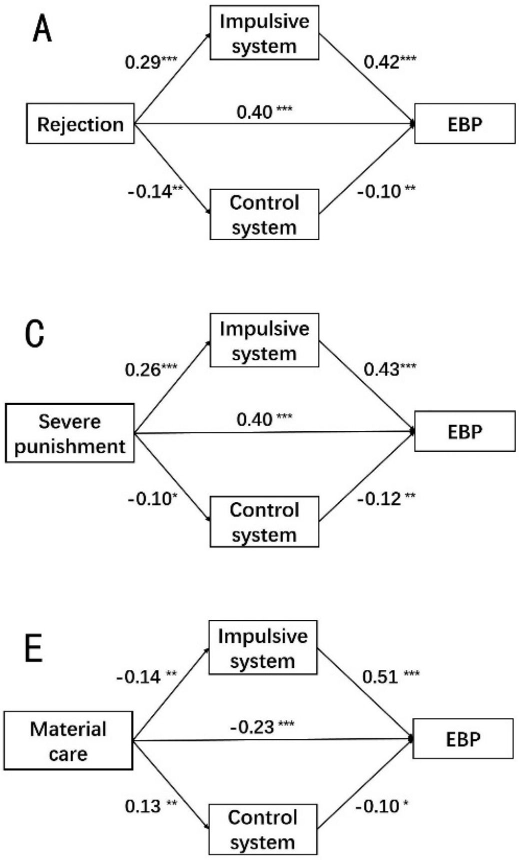

Figure S1. The mediating effects model for impulsive system and control system. (**A**–**E**) show the effects of rejection, partiality, severe punishment, emotional care and material care on EBP, respectively. Standardized path coefficients (β) are presented.

Note: *: $p < 0.05$; **: $p < 0.01$; ***: $p < 0.001$
